# Supplementary material for: National Development in the Use of Inhaled Corticosteroid Treatment in Chronic Obstructive Pulmonary Disease: Repeated Cross-Sectional Studies from 1998 to 2018
Source: Biomedicines. 2024 Feb 5;12(2):372. doi: 10.3390/biomedicines12020372 (PMC10886715; doi:10.3390/biomedicines12020372)
Supplement: Supplementary file 1 [file biomedicines-12-00372-s001.zip › biomedicines-2849885-supplementary.pdf]

Supplementary Materials

National development in the use of inhaled corticosteroid treatment in chronic obstructive pulmonary disease: repeated cross-sectional studies from 1998 to 2018

Table of contents

Inhaled corticosteroid treatment calculations (Table S1, Figure S1, Figure S2) p.2

Yearly proportion of patients in Charlson Comorbidity Index groups 1998-2018 (Figure S3) p.3

Yearly study population characteristics 1998-2018 (Table S2) p.4

Yearly proportion of patients in inhaled corticosteroid treatment groups 1998-2018 (Table S3) p.5

Inhaled corticosteroid treatment calculations

Figure S1. Formula for calculating the dose-equivalence estimation coefficient for standard particle CFC-free beclomethasone (bcm.) and the comparator ICS (comp.) from the NICE ICS dose chart.

$$\frac{\frac{\text{Mean comp. boundry, Low}}{\text{Mean bcm. boundry, Low}} + \frac{\text{Mean comp. boundry, Medium}}{\text{Mean bcm. boundry, Medium}} + \frac{\text{Mean comp. boundry, High}}{\text{Mean bcm. boundry, High}}}{3}$$

Table S1. Resulting equivalence coefficients for comparable doses.

Step	Coefficient	ATC-codes
CFC-free, standard particle beclomethasone	1*bcm	R03BA01, R03AK08, R03AL08 (AND varenummer 40417, 154147, 443228, 95456)
CFC-free, extra-fine particle beclomethasone	2.36*bcm	R03BA01, R03AK08, R03AL08 (EXCEPT varenummer 40417, 154147, 443228, 95456)
Budesonide	1.18*bcm	R03BA02, R03AK07
Ciclesonide	2.95*bcm	R03BA08
Fluticasone prop.	2.00*bcm	R03BA06, R03AK11, R03AK06, R03BA05
Fluticasone furoate	8.00*bcm	R03BA09, R03AK10, R03AL09
Mometasone furoate	1.92*bcm	R03BA07

Figure S2. Formula for the calculation of daily average exposure for the individual patient.

$$\frac{\sum(\text{Redeemed mcg ICS} * \text{Redeemed number of packets} * \text{Equivalence coefficient}) * 0{,}8}{\text{Days in study period}}$$

0.8 represents an adjustment coefficient for study assumptions of daily averaging such as continuous ICS use, correct use of redeemed doses, and no unused doses.

Figure S3. Proportion of patients according to Charlson Comorbidity Index groups for all years of the study period.

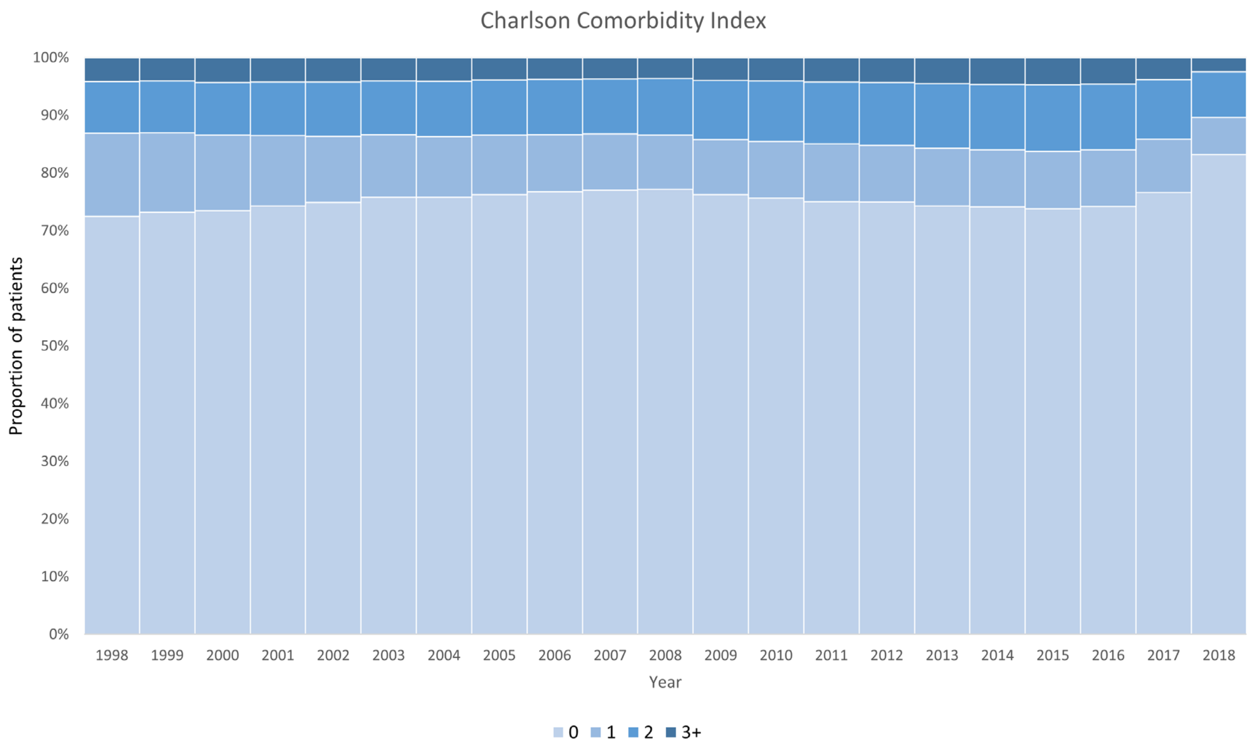

Table S2. Yearly study population characteristics 1998-2018.

	1998		1999		2000		2001		2002		2003		2004		2005		2006		2007		2008	
**Population (N)**	35,565		40,771		45,504		50,868		55,588		59,666		63,759		67,584		71,187		74,552		77,314	
	**n**	**%**	**n**	**%**	**n**	**%**	**n**	**%**	**n**	**%**	**n**	**%**	**n**	**%**	**n**	**%**	**n**	**%**	**n**	**%**	**n**	**%**
**Sex**																						
Male	16,716	47.0	19,057	46.7	21,294	46.8	23,680	46.6	25,773	46.4	27,422	46.0	29,201	45.8	30,968	45.8	32,512	45.7	34,052	45.7	35,348	45.7
Female	18,849	53.0	21,714	53.3	24,210	53.2	27,188	53.4	29,815	53.6	32,244	54.0	34,558	54.2	36,616	54.2	38,675	54.3	40,500	54.3	41,966	54.3
**Age. mean (SD)**	69	(10.5)	69	(10.6)	69	(10.7)	69	(10.7)	69	(10.8)	70	(10.8)	70	(10.8)	70	(10.9)	70	(10.9)	70	(10.9)	70	(10.9)
**Age group**																						
40-49	1,602	4.5	1,837	4.5	1,988	4.4	2,230	4.4	2,441	4.4	2,541	4.3	2,521	4.0	2,568	3.8	2,545	3.6	2,631	3.5	2,641	3.4
50-59	5,090	14.3	6,033	14.8	6,884	15.1	7,787	15.3	8,572	15.4	9,124	15.3	9,640	15.1	10,153	15.0	10,532	14.8	10,877	14.6	11,125	14.4
60-69	10,243	28.8	11,473	28.1	12,419	27.3	13,711	27.0	14,687	26.4	15,895	26.6	17,081	26.8	18,118	26.8	19,193	27.0	20,395	27.4	21,409	27.7
70-79	12,895	36.3	14,721	36.1	16,286	35.8	18,021	35.4	19,733	35.5	21,003	35.2	22,054	34.6	23,064	34.1	24,029	33.8	24,760	33.2	25,287	32.7
80+	5,735	16.1	6,707	16.5	7,927	17.4	9,119	17.9	10,155	18.3	11,103	18.6	12,463	19.5	13,681	20.2	14,888	20.9	15,889	21.3	16,852	21.8
**Charlson Comorbidity Index**																						
0	25,790	72.5	29,842	73.2	33,422	73.4	37,796	74.3	41,619	74.9	45,206	75.8	48,317	75.8	51,550	76.3	54,649	76.8	57,410	77.0	59,668	77.2
1	5,130	14.4	5,620	13.8	5,959	13.1	6,189	12.2	6,378	11.5	6,488	10.9	6,720	10.5	6,945	10.3	7,043	9.9	7,278	9.8	7,245	9.4
2	3,170	8.9	3,655	9.0	4,154	9.1	4,745	9.3	5,223	9.4	5,577	9.3	6,124	9.6	6,464	9.6	6,806	9.6	7,133	9.6	7,590	9.8
3+	1,475	4.1	1,654	4.1	1,969	4.3	2,138	4.2	2,368	4.3	2,395	4.0	2,598	4.1	2,625	3.9	2,689	3.8	2,731	3.7	2,811	3.6

	2009		2010		2011		2012		2013		2014		2015		2016		2017		2018	
**Population (N)**	80,226		83,633		87,066		89,876		92,509		95,310		97,781		99,977		100,843		99,057	
	**n**	**%**	**n**	**%**	**n**	**%**	**n**	**%**	**n**	**%**	**n**	**%**	**n**	**%**	**n**	**%**	**n**	**%**	**n**	**%**
**Sex**																				
Male	36,592	45.6	38,226	45.7	39,792	45.7	41,013	45.6	42,267	45.7	43,492	45.6	44,728	45.7	45,862	45.9	46,290	45.9	45,396	45.8
Female	43,634	54.4	45,407	54.3	47,274	54.3	48,863	54.4	50,242	54.3	51,818	54.4	53,053	54.3	54,115	54.1	54,553	54.1	53,661	54.2
**Age. mean (SD)**	70	(10.9)	70	(10.9)	71	(10.9)	71	(10.9)	71	(10.8)	71	(10.8)	71	(10.7)	71	(10.6)	72	(10.5)	72	(10.4)
**Age group**																				
40-49	2,755	3.4	2,744	3.3	2,725	3.1	2,683	3.0	2,575	2.8	2,516	2.6	2,355	2.4	2,167	2.2	1,946	1.9	1,674	1.7
50-59	11,410	14.2	11,777	14.1	12,154	14.0	12,400	13.8	12,417	13.4	12,441	13.1	12,271	12.5	12,109	12.1	11,872	11.8	11,239	11.3
60-69	22,395	27.9	23,621	28.2	24,714	28.4	25,417	28.3	26,150	28.3	26,655	28.0	27,061	27.7	27,224	27.2	27,072	26.8	26,170	26.4
70-79	25,999	32.4	26,649	31.9	27,603	31.7	28,470	31.7	29,848	32.3	31,161	32.7	32,880	33.6	34,454	34.5	35,572	35.3	35,745	36.1
80+	17,667	22.0	18,842	22.5	19,870	22.8	20,906	23.3	21,519	23.3	22,537	23.6	23,214	23.7	24,023	24.0	24,381	24.2	24,229	24.5
**Charlson Comorbidity Index**																				
0	61,181	76.3	63,283	75.7	65,341	75.0	67,356	74.9	68,685	74.2	70,678	74.2	72,139	73.8	74,217	74.2	77,270	76.6	82,400	83.2
1	7,668	9.6	8,187	9.8	8,706	10.0	8,825	9.8	9,319	10.1	9,428	9.9	9,770	10.0	9,821	9.8	9,323	9.2	6,427	6.5
2	8,184	10.2	8,788	10.5	9,329	10.7	9,810	10.9	10,367	11.2	10,784	11.3	11,244	11.5	11,368	11.4	10,386	10.3	7,779	7.9
3+	3,193	4.0	3,375	4.0	3,690	4.2	3,885	4.3	4,138	4.5	4,420	4.6	4,628	4.7	4,571	4.6	3,864	3.8	2,451	2.5

Table S3. Yearly proportion of patients in inhaled corticosteroid treatment groups 1998-2018.

	**1998**		**1999**		**2000**		**2001**		**2002**		**2003**		**2004**		**2005**		**2006**		**2007**		**2008**	
**Population (N)**	35,565		40,771		45,504		50,868		55,588		59,666		63,759		67,584		71,187		74,552		77,314	
	**n**	**%**	**n**	**%**	**n**	**%**	**n**	**%**	**n**	**%**	**n**	**%**	**n**	**%**	**n**	**%**	**n**	**%**	**n**	**%**	**n**	**%**
**ICS treatment**																						
No ICS	17,986	50.6	20,755	50.9	24,120	53.0	27,087	53.2	29,584	53.2	30,987	51.9	31,930	50.1	33,058	48.9	34,094	47.9	34,912	46.8	35,734	46.2
Low dose	4,009	11.3	4,583	11.2	5,161	11.3	5,997	11.8	6,978	12.6	8,344	14.0	9,713	15.2	11,183	16.5	12,171	17.1	13,696	18.4	14,612	18.9
Medium dose	7,508	21.1	8,636	21.2	9,493	20.9	10,334	20.3	11,194	20.1	11,992	20.1	13,004	20.4	14,006	20.7	15,226	21.4	16,100	21.6	16,639	21.5
High dose	6,053	17.0	6,797	16.7	6,730	14.8	7,444	14.6	7,830	14.1	8,332	14.0	9,102	14.3	9,323	13.8	9,687	13.6	9,835	13.2	10,319	13.3

	**2009**		**2010**		**2011**		**2012**		**2013**		**2014**		**2015**		**2016**		**2017**		**2018**	
**Population (N)**	80,226		83,633		87,066		89,876		92,509		95,310		97,781		99,977		100,843		99,057	
	**n**	**%**	**n**	**%**	**n**	**%**	**n**	**%**	**n**	**%**	**n**	**%**	**n**	**%**	**n**	**%**	**n**	**%**	**n**	**%**
**ICS treatment**																				
No ICS	36,955	46.1	38,728	46.3	41,110	47.2	43,280	48.2	45,451	49.1	48,435	50.8	51,129	52.3	54,203	54.2	56,654	56.2	57,072	57.6
Low dose	15,208	19.0	15,671	18.7	15,917	18.3	15,841	17.6	15,975	17.3	15,665	16.4	15,639	16.0	15,595	15.6	15,492	15.4	14,782	14.9
Medium dose	17,392	21.7	18,255	21.8	18,961	21.8	19,328	21.5	19,675	21.3	19,862	20.8	20,234	20.7	20,283	20.3	19,614	19.5	17,931	18.1
High dose	10,670	13.3	10,979	13.1	11,078	12.7	11,427	12.7	11,408	12.3	11,348	11.9	10,779	11.0	9,896	9.9	9,083	9.0	9,272	9.4

* Observations with missing information are not included in the ICS distribution. Therefore, not always sum = 100%.
